# Evaluation of the accuracy of mammography, ultrasound and magnetic resonance imaging in suspect breast lesions

**DOI:** 10.6061/clinics/2020/e1805

**Published:** 2020-07-15

**Authors:** Renato de Oliveira Pereira, Larissa Almondes da Luz, Diego Cipriano Chagas, Jefferson Rodrigues Amorim, Elmo de Jesus Nery-Júnior, Araci Castelo Branco Rodrigues Alves, Flávio Teixeira de Abreu-Neto, Maria da Conceição Barros Oliveira, Danylo Rafhael Costa Silva, José Maria Soares-Júnior, Benedito Borges da Silva

**Affiliations:** I Programa de Pos-Graduacao em Ciencias da Saude, Universidade Federal do Piaui, Teresina, PI, BR; II Programa de Pos-Graduacao, Rede Nordeste de Biotecnologia (RENORBIO), Universidade Federal do Piaui, Teresina, PI, BR; III Departamento de Mastologia, Hospital Getulio Vargas, Teresina, PI, BR; IV Disciplina de Ginecologia, Departamento de Obstetricia e Ginecologia, Hospital das Clinicas HCFMUSP, Faculdade de Medicina, Universidade de Sao Paulo, Sao Paulo, SP, BR

**Keywords:** Breast Cancer, Mammography, Ultrasonography, Magnetic Resonance Imaging

## Abstract

**OBJECTIVES:**

In breast cancer diagnosis, mammography (MMG), ultrasonography (USG) and magnetic resonance imaging (MRI) are the imaging methods most used. There is a scarcity of comparative studies that evaluate the accuracy of these methods in the diagnosis of breast cancer.

**METHODS:**

A cross-sectional study was carried out through the review of electronic medical records of 32 female patients who underwent breast imaging examinations at a imaging diagnostic center in Teresina, State of Piauí, Brazil. Patients who had these three imaging methods at the time of the evaluation of the same nodule were included. The nodule must have been classified as suspect by the BI-RADS^®^ system in at least one of the methods. Data from each method were compared with the histopathological examination. Statistical analysis used the calculation of proportions in Excel 2010.

**RESULTS:**

MMG showed 56.2%, 87.5%, 81.8%, 66.7% and 71.8% of sensitivity, specificity, positive predictive value (PPV), negative predictive value (NPV) and accuracy, respectively. USG had 75%, 18.8%, 48%, 42.8% and 46.9% of sensitivity, specificity, PPV, NPV and accuracy, respectively. In turn, MRI had 100%, 50%, 66.7%, 100% and 75% of sensitivity, specificity, PPV, NPV and accuracy, respectively.

**CONCLUSION:**

Thus, MRI and MMG were more accurate in evaluating suspicious breast lumps. MRI had a low specificity, mainly to high breast density, while MMG had also sensitivity limited due to high breast density and USG has been proven to be useful in these patients.

## INTRODUCTION

Breast cancer is the most common cancer among the female population worldwide, with an incidence of 2,088,849 new cases in 2018 and mortality of 626,679 in this year (1). In Brazil, breast cancer is the most common malignant cancer in women after non-melanoma skin cancer, with approximately 59,700 new cases in 2019 and 15,403 deaths registered in 2015 (2).

The success of breast cancer treatment depends on the early diagnosis administering treatment during the initial stage of the disease, which has an influence on overall survival, regardless of the advancements in therapy (3,4). The main imaging procedures that usually lead to an early detection of breast cancer are mammography, ultrasonography and magnetic resonance imaging. Mammography remains the standard examination tool in the systematic screening of breast cancer, because aside from its easy implementation and standardization, it has the advantage of allowing revisions and a direct comparison with mammograms from previous studies (3).

However, breast USG remains the first choice for the characterization of masses detected in the MMG or as an adjunct screening method (6). The ability of USG to visualize small cancers that are clinically and mammographically hidden in women with dense breasts has already been proven (5). Of all the currently available breast imaging techniques, MRI has the highest sensitivity for the diagnosis of invasive breast cancer, and this sensitivity is not impaired by the amount or density of fibroglandular tissue, fibrous scarring, radiotherapy, breast implants, or other types of breast reconstruction. Nevertheless, this method has some limitations that prohibit its routine use, such as the higher cost, lack of standardization in acquisition techniques, and interpretation guidelines, as well as controversies about its low specificity with high rate of false positive results (7).

Therefore, the importance of these imaging methods in the diagnosis of suspected breast tumors and the scarcity of studies comparing the accuracy of mammography, ultrasound and magnetic resonance imaging, served as the basis for the design of the present study.

## PATIENTS AND METHODS

### Patients

This study included the imaging findings of 40 women aged 36–78 years with suspected breast tumors through at least one of the methods (MMG, USG, and MRI) at the Imaging Diagnosis Unit together with the Mastology Clinic of the Getúlio Vargas Hospital, Federal University of Piauí between January and December 2018. Eight women were excluded due to inconclusive histopathological results. The Internal Review Board of the Federal University of Piauí approved the study and patient data had been archived since 2010.

## METHODS

The study was approved by the Ethic Committee in Research of Federal University under number CAAE 03877118.4.0000.5214.

The 32 patients selected had a BI-RADS^®^ 4 or 5 suspicious lesion in at least one of the three imaging methods. From these results the sensitivity, specificity, positive predictive value (PPV), negative predictive value (NPV) and accuracy with area under the ROC curve of each one of the imaging methods (MMG, USG, and MRI) were evaluated. The images as well as the histopathological examination of the breast lesion were performed by the same professional specialist in each area.

The data obtained were stored in an electronic database created in the Excel 2010 program (Windows 10, Microsoft Corporation, Random, WA, USA). Statistical analysis involving the calculation of sample proportions was also performed in Excel 2010.

## RESULTS

The study involved 32 patients with suspected breast lesions detected by at least one of the imaging methods described above, in which a correlation between the imaging findings and histopathological examination of the lesion was performed. Of the 32 patients, 16 were diagnosed with breast cancer and 16 exhibited a benign histopathology outcome. The age of the patients ranged between 36 and 78 years, the mean age being 54.6 years. The average size of the lesions was 1.6 cm, ranging between 0.5 and 4.1 cm (Table 1).

There was concordance between the mammography and the histopathological examination results in 23 of 32 patients. These 23 patients comprised of 9 malignant cases and 14 non-suspect cases that were benign upon histopathology examination. There was disagreement in 9 cases, where 2 cases were considered suspect cases with MMG but benign upon histopathology analysis and 7 were considered non-suspect cases with MMG but malignant upon histopathological examination. The sensitivity, specificity, PPV, NPV, and accuracy of MMG were 56.2%, 87.5%, 81.8%, 66.7%, and 78.1%, respectively, and an area under the receiver operating characteristic (ROC) curve of 0.728, all within the confidence interval (Table 2).

With regards to USG, there was agreement in 15 of 32 patients. Among the 15 patients, 12 suspect cases were malignant and 3 non-suspect cases were benign upon histopathology. There were disagreements in 17 cases, with 13 considered suspect cases with USG but benign upon histopathology and 4 considered non-suspect cases with USG but malignant upon histopathological examination. The sensitivity, specificity, PPV, NPV, and accuracy were 75%, 18.8%, 48.0%, 42.8% and 46.9%, respectively, and an area under the ROC curve of 0.500, all within the confidence interval (Table 3).

MRI agreed with the histopathological diagnosis in 24 out of 32 cases. The 24 cases comprised of 16 suspect cases which were malignant and 8 non-suspect cases which were benign upon histopathology. There was disagreement in 8 cases, all of which were suspect cases with MRI but benign upon histopathology. The sensitivity, specificity, PPV, NPV, and accuracy of MRI was 100%, 50%, 66.7%, 100%, and 75%,respectively, and an area under the ROC curve of 0.500, all within the confidence interval (Table 4).

## DISCUSSION

Among the methods used, MRI displayed the highest sensitivity, while MMG displayed the lowest sensitivity. Breast density is one of the most important factors in the concealment of malignant lesions with mammography, since the superposition of fibroglandular tissue may obscure small tumors and eventually even considerable masses (8). Another limiting factor is the size of the tumor, because the smaller the lesion, the less likely it is to detected (9). Of the seven false-negative cases of MMG in this study, in six cases the lesion was not visualized with the method and of these, four had dense breasts and had a small lesion. In one case the lesion was seen and classified as benign, also in a patient with dense breast and small lesion.

Incidentally, USG detected all the lesions not shown by MMG in dense breasts. However, the factors that interfere with the ability of USG to detect cancer include the correlation with the mammographic study, technical parameters optimized for the USG and characteristics of the patient and the lesion, with large and fatty breasts and small lesions, deep and heterogeneous echogenicity impeding the diagnosis (8,10). In our study, of the 16 cases of histologically confirmed cancer, USG did not identify four, due to the small size of the lesion in 3 cases and deep location in 1 case. In contrast, MRI scan showed no false-negative results, regardless of breast density or the size of the lesion, which proved to be limiting factors in the other two imaging modalities.

With regards to specificity, MMG showed better results, because it had only two false-positive cases which were classified as BI-RADS^®^ 4, while in the classification 5 which has a greater degree of suspicion, there were no false-positive results (11). A false-positive result in the mammogram depends on several factors, such as previous examinations for comparison, experience of the radiologist, surgical scars and increased breast density (12), which concurs with the two false-positive cases in this study that occurred in dense breasts.

Furthermore, in this study, USG showed less specificity. Of the 13 false positive cases, 12 cases were classified as BI-RADS^®^ 4 and only 1 classified as 5, although this last case had a probability greater than 95% of malignancy, which can be explained by the sample size of the study. In addition, in 10 of the 13 false-positive cases in USG, the lesions were small. Previously, Abdullah et al. (13) observed a low interobserver concordance in the ultrasonographic characterization of small breast lesions, which can explain the false results.

Although not presenting a specificity as low as the USG, MRI results in eight false positive results, all classified as BI-RADS^®^ 4. The specificity of MRI in the literature is variable, with studies reporting values between 37% and 97% (14). The meta-analysis of Zhang and Ren (15) found a combined specificity of 70% for MRI in the diagnosis of breast cancer. This potentially limited specificity has been attributed to the fact that many benign lesions and normal breast tissue may be highlighted with the paramagnetic contrast medium, demonstrating overlap with malignant lesions, both in kinetic and morphological terms (16). On the other hand, some authors have suggested that MRI of the breast does not increase the rate of false positive biopsies, with a specificity similar to MMG but significantly greater than that of USG (7). Despite the controversy, in our study, of the eight false-positive cases, six had dense breasts, which may have limited the specificity of MRI. In the study by Boné et al. (17), increased breast density also proved to be a limiting factor for the MRI, reducing the specificity of the method, probably due to a higher rate of proliferative alterations found in this type of breast cancer.

In summary, the present study showed MRI and MMG were more accurate in evaluating suspicious breast lumps. MRI had a low specificity, mainly in high breast density, while MMG had also sensitivity limited due to high breast density and USG has been proven to be useful in these patients. Regardless, further large-scale studies are needed to compare these methods.

## AUTHOR CONTRIBUTIONS

Pereira RO, Silva BB and Soares-Junior JM participated in all stages of the work. Luz LA contributed in the manuscript writing. Chagas DC, Amorim JR, Alves ACBR, Abreu-Neto FT, Nery-Júnior EJ, Oliveira MCB and Silva DRC contributed to the data collection and analysis. All authors read and approved the final manuscript.

## Figures and Tables

**Table 1:** General characteristics of studied patients.

Characteristic	Mean
Age (years)	54.6 (36-78)
Breast lump size (cm)	1.6 (0.5-4.1)

Source: Research data.

**Table 2:** Comparison between mammography (MMG) and histopathology of suspicious breast lumps in at least one of the methods studied.

MMG \ Histopathology	Malignant	Non-malignant	Total
Suspicious	9	2	11
Non-suspicious	7	14	21
Total	16	16	32

Sensitivity 56.2%; Specificity 87.5%; NPV 66.7%; PPV 81.8%; Accuracy 71.8%; Area under the receiver operating characteristic curve 0.712.

**Table 3:** Comparison between ultrasound (USG) and histopathology of suspicious breast lumps in at least one of the methods studied.

USG \ Histopathology	Malignant	Non-malignant	Total
Suspicious	12	13	25
Non-suspicious	4	3	27
Total	16	16	32

Sensitivity 75% / Specificity 18.8% / NPV 42.8% / PPV 48% / Accuracy 46.9% / Area under the receiver operating characteristic curve 0.500.

**Table 4:** Comparison between magnetic resonance imaging (MRI) and histopathology of suspicious breast lumps in at least one of the methods studied.

MRI \ Histopathology	Malignant	Non-malignant	Total
Suspicious	16	8	24
Non-suspicious	0	8	8
Total	16	16	32

Sensitivity 100% / Specificity 50% / NPV 100% / PPV 66.7% / Accuracy 75% / Area under the receiver operating characteristic curve 0.750.
